# Higher body roundness index (BRI) increases infertility among U.S. women aged 18–45 years

**DOI:** 10.1186/s12902-024-01799-8

**Published:** 2024-12-18

**Authors:** Hongyang Gong, Shuqin Duan, Seok Choi, Shaoqun Huang

**Affiliations:** 1 Department of Oncology Surgery, Fuzhou Hospital of Traditional Chinese Medicine Affiliated to Fujian University of Traditional Chinese Medicine, No.102 Gudong Road, Gulou District, Fuzhou City, Fujian Province 350001 China; 2 Department of Physiology, College of Medicine, Chosun University, Gwangju, Korea; 3 Graduate School of Jilin University, Changchun City, China

**Keywords:** Infertility, Body roundness index, NHANES, U.S. women, Association

## Abstract

**Objective:**

Infertility is associated with obesity. The Body Roundness Index (BRI) is a body measurement index related to obesity that more accurately assesses body and visceral fat levels. However, the relationship between BRI and infertility remains unclear. Therefore, this study aims to determine the relationship between BRI and infertility.

**Methods:**

This study utilized data from the National Health and Nutrition Examination Survey (NHANES) from 2013 to 2018 and included 3,528 women aged 18–45 years. Multivariate logistic regression was employed to investigate the association between BRI and infertility. Restricted cubic spline (RCS) analysis explored the linear or non-linear relationship between BRI and infertility. Interaction analyses were conducted on subgroups to validate the findings. To verify the robustness of the results, we performed several sensitivity analyses, including propensity score matching(PSM) and multiple imputations for missing data. Furthermore, the predictive capabilities of various anthropometric indices—including BRI, weight-adjusted waist index (WWI), body mass index (BMI), and weight—on infertility incidence were assessed using Receiver Operating Characteristic (ROC) curve analysis.

**Results:**

There was a significant positive association between BRI and infertility. After adjusting for covariates, for each unit increase in BRI, there was a 12% increase in the probability of infertility (*P* < 0.001). This positive correlation persisted when BRI was categorized into quartiles. Moreover, as BRI increased, there was a trend towards higher infertility prevalence (*P for trend* < 0.001). The dose-response curve indicated a linear association between BRI and infertility, with higher BRI associated with higher infertility risk. The correlation between BRI and infertility persisted in subgroup analysis and multiple imputations. The ROC curve analysis revealed that BRI had a superior predictive capability compared to traditional obesity indices, with an area under the curve (AUC) of 0.618 (95% CI, 0.588–0.648).

**Conclusion:**

The results of this study show a strong positive correlation between BRI and the prevalence of infertility.

**Clinical trial number:**

Not Applicable.

**Supplementary Information:**

The online version contains supplementary material available at 10.1186/s12902-024-01799-8.

## Introduction

Infertility is defined as the inability of a woman to conceive after at least 12 months of unprotected intercourse. This condition is a widespread global issue. The age-standardized prevalence rate of female infertility worldwide increased by 14.962%, from 1,366.85 per 100,000 women in 1990 to 1,571.35 per 100,000 women in 2017 [1]. Infertility is a reproductive disorder caused by various etiologies, posing a significant social burden on women [2]. Therefore, exploring the preventable and modifiable factors of infertility and providing support for its management is crucial.

Obesity is a multifactorial disease, and currently, nearly one-third of the global population is classified as overweight or obese. Body Mass Index (BMI) is commonly used to measure obesity. BMI is calculated by dividing an individual’s weight in kilograms by the square of their height in meters. For adults, current guidelines from the Centers for Disease Control and Prevention (CDC) and the World Health Organization (WHO) define a normal BMI range as 18.5 to 24.9. A BMI of 25 kg/m² or higher is considered overweight, while a BMI of 30 kg/m^²^ or higher is classified as obese [3]. Numerous studies have shown that obesity is closely associated with diabetes [4], cardiovascular diseases [5], and cancer incidence [6]. Despite its widespread use, BMI does not distinguish between fat-free mass (FFM) and fat mass (FM). To better understand the relationship between body fat and visceral adipose tissue, the Body Roundness Index (BRI) was proposed in 2013 [7]. Compared to subcutaneous fat accumulation, which is linked to increased tendencies for hyperglycemia, hyperinsulinemia, and hypertriglyceridemia, all characteristics of insulin resistance syndrome [8], BRI may provide a more advantageous predictive measure than BMI. Research has indicated that BRI has greater predictive value for colorectal cancer [9], cardiovascular diseases (CVD) [10], diabetes among hypertensive populations [11], and type 2 diabetes [12].

The relationship between obesity and infertility is well-established [13]. In recent years, there has been increasing attention on visceral obesity [14]. Obesity can be measured using various indices, but BMI alone is insufficient as a marker for abdominal obesity [15]. The Body Roundness Index (BRI), which reflects abdominal obesity, has not yet been studied for infertility. This study aims to explore the correlation between BRI and the risk of infertility using large sample data from the National Health and Nutrition Examination Survey (NHANES) 2013–2018 and to investigate the potential mechanisms involved.

## Methods

### Study population

**Fig. 1:**
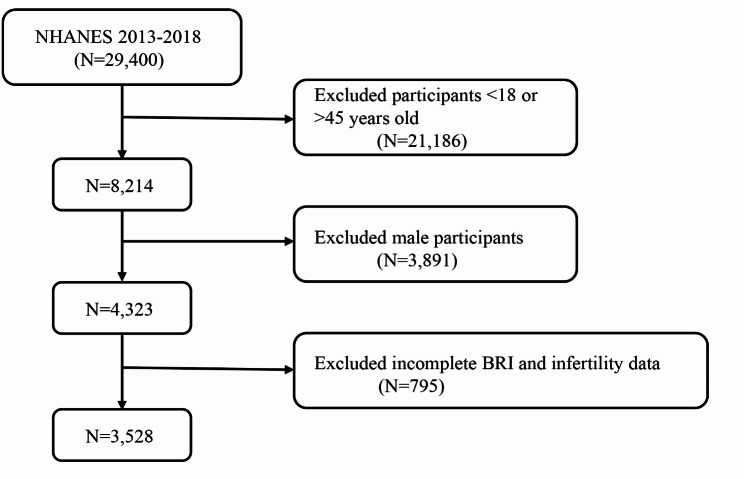
A flow diagram of eligible participant selection in the National Health and Nutrition

The National Health and Nutrition Examination Survey (NHANES) is a nationally representative cross-sectional survey conducted through home interviews and mobile examination centers, aimed at assessing the health and nutritional status of the U.S. population. This survey utilized data from 29,400 participants over three cycles of NHANES, spanning from 2013 to 2018. After excluding individuals < 18 or > 45 years old (*n* = 21,186), males (*n* = 3,891), and participants with missing or incomplete BRI and infertility data (*n* = 795), a total of 3,528 participants were included in the final analysis. Figure 1 displays a flowchart of the entire selection process. NHANES is approved by the Research Ethics Review Board of the National Center for Health Statistics, and all participants provided informed consent [16]. The data used in this study are de-identified and publicly available (https://www.cdc.gov/nchs/nhanes/index.htm).

### Measurement

#### Assessment of BRI

BRI is a novel body shape assessment index, calculated using participants’ height (cm) and waist circumference (cm) [17]. The specific calculation formula is as follows:

$$\:BRI=364.2-365.5\times\:\sqrt{1-{\frac{\left(\frac{waist\:circumference}{2\pi\:}\right)}{{(0.5\times\:height)}^{2}}}^{2}}$$.

#### Diagnosis of infertility

According to previous research [18, 19], infertility is defined as a reduction in an individual or their partner’s ability to conceive, characterized by the inability to become pregnant after one year or more of regular, unprotected intercourse. In this study, the assessment of infertility is derived from the NHANES Reproductive Health Questionnaire (RHQ074). Specifically, participants were asked the following question: “Have you ever tried to get pregnant for a year or longer without becoming pregnant?” Participants who answered “yes” were classified as infertile (https://wwwn.cdc.gov/Nchs/Nhanes/2013-2014/RHQ_H.htm#RHQ074).

### Covariables

According to previous studies [18, 19], the covariates in this research include age, race, marital status, education level, family poverty-to-income ratio (PIR), smoking, alcohol consumption, hypertension, diabetes, and hypercholesterolemia. For detailed information on these covariates, please refer to Table S1.

### Statistical analyses

In this study, all data were statistically analyzed using R (version 4.3.1). The data were weighted, with continuous variables presented as mean ± standard deviation, and *p*-values calculated using weighted linear regression models. Percentages for categorical variables (weighted N, %) and their *p*-values were calculated using weighted chi-square tests. The association between BRI and infertility was analyzed using multivariable logistic regression models, where BRI was categorized into quartiles. Trend tests and *p*-values for linear trends were calculated to determine the consistency of the relationship. Three models were constructed in this study: (1) an unadjusted crude model; (2) a model adjusted for age, race, education level, marital status, and family poverty-to-income ratio (PIR); and (3) a model further adjusted for smoking, alcohol consumption, hypertension, diabetes, and hypercholesterolemia. A smooth curve fitting was applied to further explore the potential linear relationship between BRI and infertility. Additionally, odds ratios (ORs) were calculated for every 1-unit increase in BRI, with subgroup analyses conducted based on age, race, marital status, education level, PIR, smoking, alcohol consumption, hypertension, diabetes, and hypercholesterolemia. Multiple imputations by chained equations (MICE) and repeated the main analyses. We used multiple imputations, based on 5 imputed data sets to account for missing baseline data [20]. Finally, to reduce selection bias and balance the distribution of covariates between the non-infertility and infertility groups, propensity score matching (PSM) was performed in a 1:1 ratio with a caliper width of 0.01 times the standard deviation of the logit of the propensity score. The significance was determined by *p*-values below 0.05.

## Results

### Characteristics of the participants

This study included 3,528 women aged 18 to 45, representing approximately 51,123,046 women of reproductive age in the United States. The prevalence of infertility was 11% (equivalent to 5,793,958 women), with a mean (SD) BRI value of 6.43 (3.08). Table 1 shows that women with infertility had a higher BRI compared to those without infertility (non-infertile: 5.24 (2.65), Infertile: 6.43 (3.08)). Significant differences were found between the infertile and non-infertile groups regarding age, cohabitation with a partner, smoking, hypertension, diabetes, and hyperlipidemia (all *p* < 0.05). The baseline after PSM is shown in Table S3.

**Table 1:** Baseline characteristics of all participants were stratified by infertility, weighted

Characteristic	Overall, *N* = 51,123,046 (100%)	Non-infertility, *N* = 45,329,088 (89%)	Infertility, *N* = 5,793,958 (11%)	*P* Value
**No. of participants in the sample**	3,528	3,164	364	**-**
**Age (%)**				**< 0.001**
*18–25*	15,030,743 (29%)	14,310,789 (32%)	719,954 (12%)	
*26–34*	16,139,528 (32%)	14,397,104 (32%)	1,742,425 (30%)	
*> 34*	19,952,775 (39%)	16,621,196 (37%)	3,331,579 (58%)	
**Race (%)**				0.100
*Non-Hispanic White*	28,635,786 (56%)	25,017,130 (55%)	3,618,655 (62%)	
*Other*	9,510,598 (19%)	8,658,404 (19%)	852,193 (15%)	
*Non-Hispanic Black*	6,845,835 (13%)	6,139,901 (14%)	705,934 (12%)	
*Mexican American*	6,130,828 (12%)	5,513,652 (12%)	617,176 (11%)	
**Married/live with partner (%)**				**< 0.001**
*No*	19,229,843 (40%)	17,919,894 (43%)	1,309,948 (23%)	
*Yes*	28,656,737 (60%)	24,205,957 (57%)	4,450,780 (77%)	
**Education level (%)**				0.588
*Below high school*	5,493,446 (11%)	4,875,470 (12%)	617,976 (11%)	
*High School or above*	42,393,134 (89%)	37,250,382 (88%)	5,142,752 (89%)	
**PIR (%)**				0.108
*Not Poor*	33,606,384 (70%)	29,431,242 (70%)	4,175,142 (75%)	
*poor*	14,072,815 (30%)	12,671,795 (30%)	1,401,020 (25%)	
**Smoking (%)**				**0.012**
*Never*	35,406,036 (69%)	31,857,251 (70%)	3,548,786 (61%)	
*Former*	6,069,938 (12%)	5,201,022 (11%)	868,916 (15%)	
*Current*	9,647,072 (19%)	8,270,815 (18%)	1,376,256 (24%)	
**Drinking (%)**				0.061
*former*	2,601,289 (5.3%)	2,094,740 (4.8%)	506,548 (9.0%)	
*heavy*	13,105,738 (26%)	11,561,989 (26%)	1,543,749 (28%)	
*mild*	12,825,776 (26%)	11,359,023 (26%)	1,466,753 (26%)	
*moderate*	13,559,212 (27%)	12,079,207 (28%)	1,480,005 (26%)	
*never*	7,429,995 (15%)	6,817,834 (16%)	612,161 (11%)	
**Hypertension (%)**				**< 0.001**
*No*	43,682,966 (85%)	39,218,707 (87%)	4,464,259 (77%)	
*Yes*	7,440,080 (15%)	6,110,381 (13%)	1,329,699 (23%)	
**Diabetes (%)**				**< 0.001**
*No*	46,014,179 (94%)	41,112,084 (94%)	4,902,095 (89%)	
*Yes*	2,984,448 (6.1%)	2,401,930 (5.5%)	582,519 (11%)	
**High cholesterol (%)**				**< 0.001**
*No*	45,007,588 (88%)	40,305,200 (89%)	4,702,388 (81%)	
*Yes*	6,115,458 (12%)	5,023,888 (11%)	1,091,570 (19%)	
**BRI (mean (SD))**	5.38 (2.73)	5.24 (2.65)	6.43 (3.08)	**< 0.001**

Mean (SD) for continuous variables: the *P* value was calculated by the weighted linear regression model

Percentages (weighted N, %) for categorical variables: the *P* value was calculated by the weighted chi-square test

Abbreviation: BRI, body roundness index; PIR, Ratio of family income to poverty

### Association between BRI and infertility

**Fig. 2:**
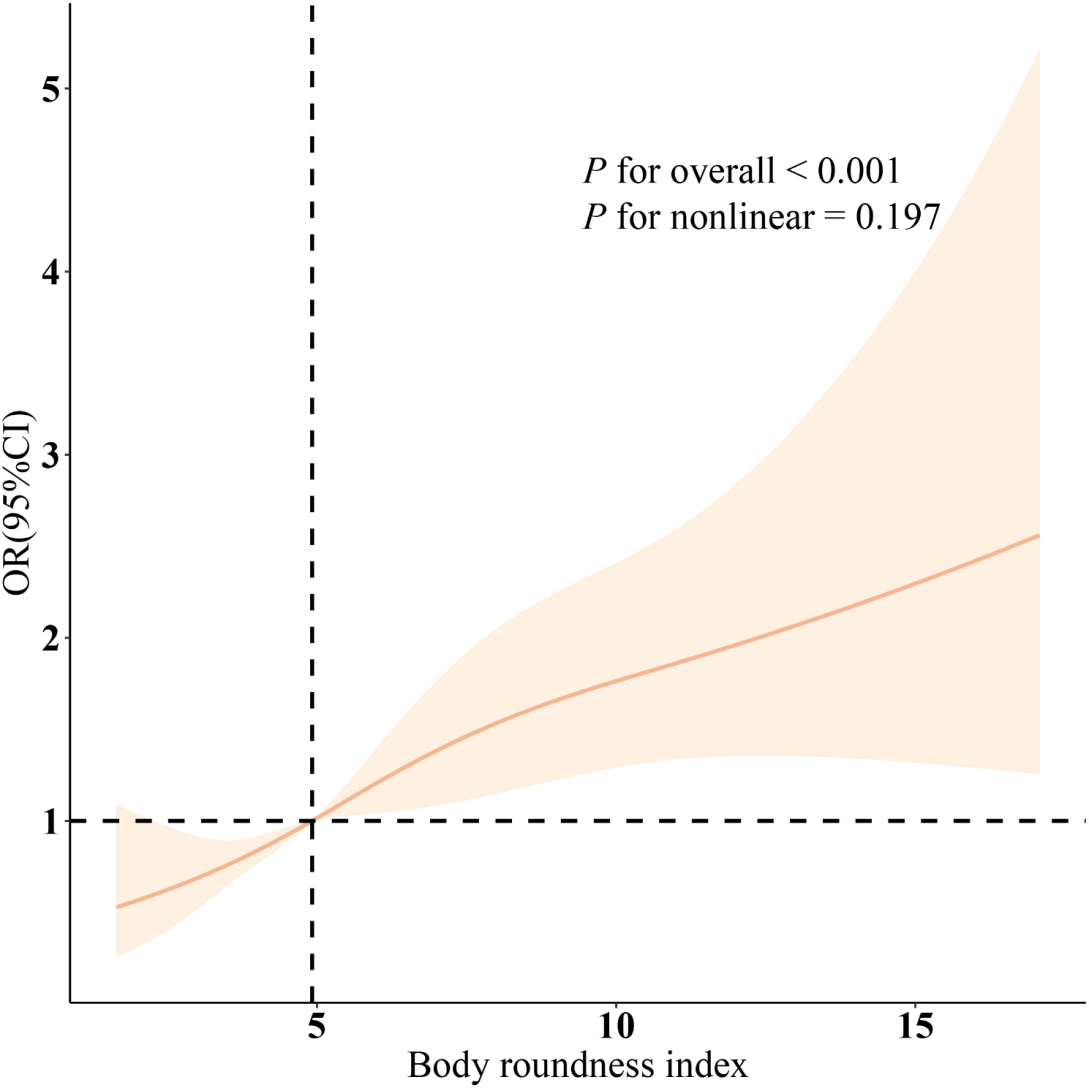
The smooth curve fitting analysis of BRI and infertility. OR (solid lines) and 95% confidence levels (shaded areas) were adjusted for age, education level, marital, PIR, race, smoking, drinking, hypertension, diabetes, and high cholesterol were adjusted. Abbreviation: BRI, body roundness index; PIR, Ratio of family income to poverty; ORs, odds ratios; CI, confidence interval

As shown in Table 2, the relationship between BRI and infertility was assessed using three models. In Model 3, after fully adjusting for covariates, each unit increase in BRI was associated with a 12% increase in the probability of infertility (OR: 1.12; 95% CI: 1.05, 1.19). The prevalence of infertility increased progressively with higher BRI quartiles (with Q1 as the reference). The corresponding results were: Q2 [Odds Ratio: 1.31; 95% CI: 0.83, 2.05], Q3 [Odds Ratio: 1.72; 95% CI: 0.98, 3.02], and Q4 [Odds Ratio: 1.60; 95% CI: 1.52, 4.44]. Additionally, there was a statistically significant trend of increasing infertility prevalence with higher BRI (P for trend < 0.001). Figure 2 further illustrates the significant positive linear relationship between BRI and infertility prevalence (overall *P* < 0.001; nonlinearity *P* = 0.468). It is important to note (Table S4) that logistic regression results did not show statistical significance after PSM, although the differences between confounders were balanced.

**Table 2:** Adjusted odds ratios (ORs) of BRI and infertility, weighted

BRI	Model 1 [OR (95% CI)]	*p*-value	Model 2 [OR (95% CI)]	*p*-value	Model 3 [OR (95% CI)]	*p*-value
Continuous (Per 1 unit increase)	1.14 (1.09, 1.20)	< 0.001	1.13 (1.07, 1.20)	< 0.001	1.12 (1.05, 1.19)	< 0.001
Quartile						
Q1	1 (ref.)		1 (ref.)		1 (ref.)	
Q2	1.48 (1.00, 2.19)	0.053	1.23 (0.79, 1.90)	0.300	1.31 (0.83, 2.05)	0.200
Q3	2.08 (1.32, 3.26)	0.002	1.71 (1.03, 2.83)	0.039	1.72 (0.98, 3.02)	0.057
Q4	3.14 (1.95, 5.06)	< 0.001	2.62 (1.51, 4.52)	0.001	2.60 (1.52, 4.44)	0.001
*P for trend*	< 0.001		0.001		0.001	

Model 1: no covariates were adjusted

Model 2: age, education level, marital, PIR, and race were adjusted

Model 3: age, education level, marital, PIR, race, smoking, drinking, hypertension, diabetes, and high cholesterol were adjusted

Abbreviation: BRI, body roundness index; PIR, Ratio of family income to poverty; ORs, odds ratios; CI, confidence interval

### Subgroup analysis

**Fig. 3:**
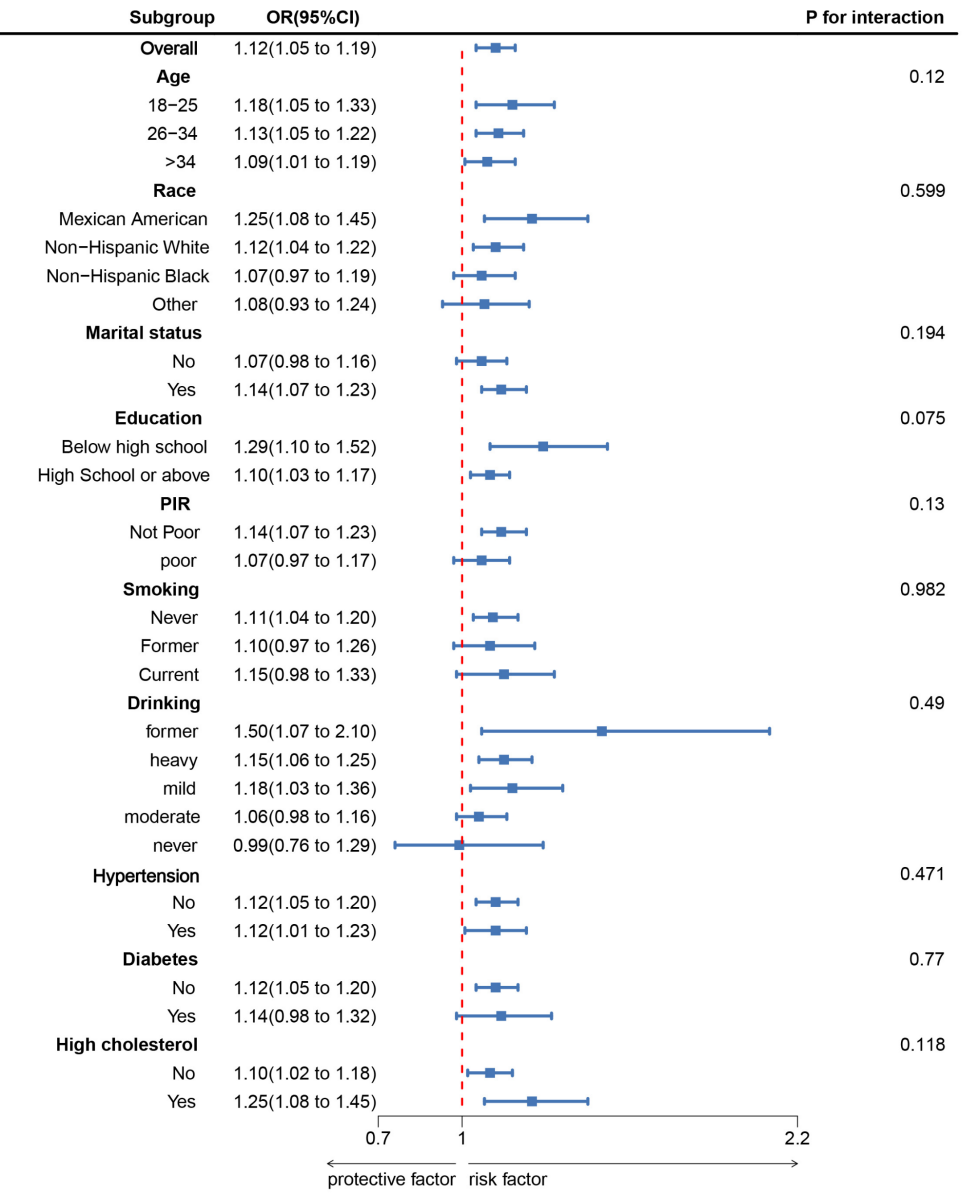
Subgroup analysis between BRI and infertility. ORs were calculated as each unit increased in BRI. Analyses were adjusted for age, education level, marital, PIR, race, smoking, drinking, hypertension, diabetes, and high cholesterol were adjusted. Abbreviation: BRI, body roundness index; PIR, Ratio of family income to poverty; ORs, odds ratios; CI, confidence interval

As shown in Fig. 3, a subgroup analysis was conducted based on age, race, marital status, education level, PIR, smoking, alcohol consumption, hypertension, diabetes, and hyperlipidemia. The results were like the main analysis, indicating no significant interaction effects (all *p*-values for interaction > 0.05).

### BRI as a predictor for infertility

**Fig. 4:**
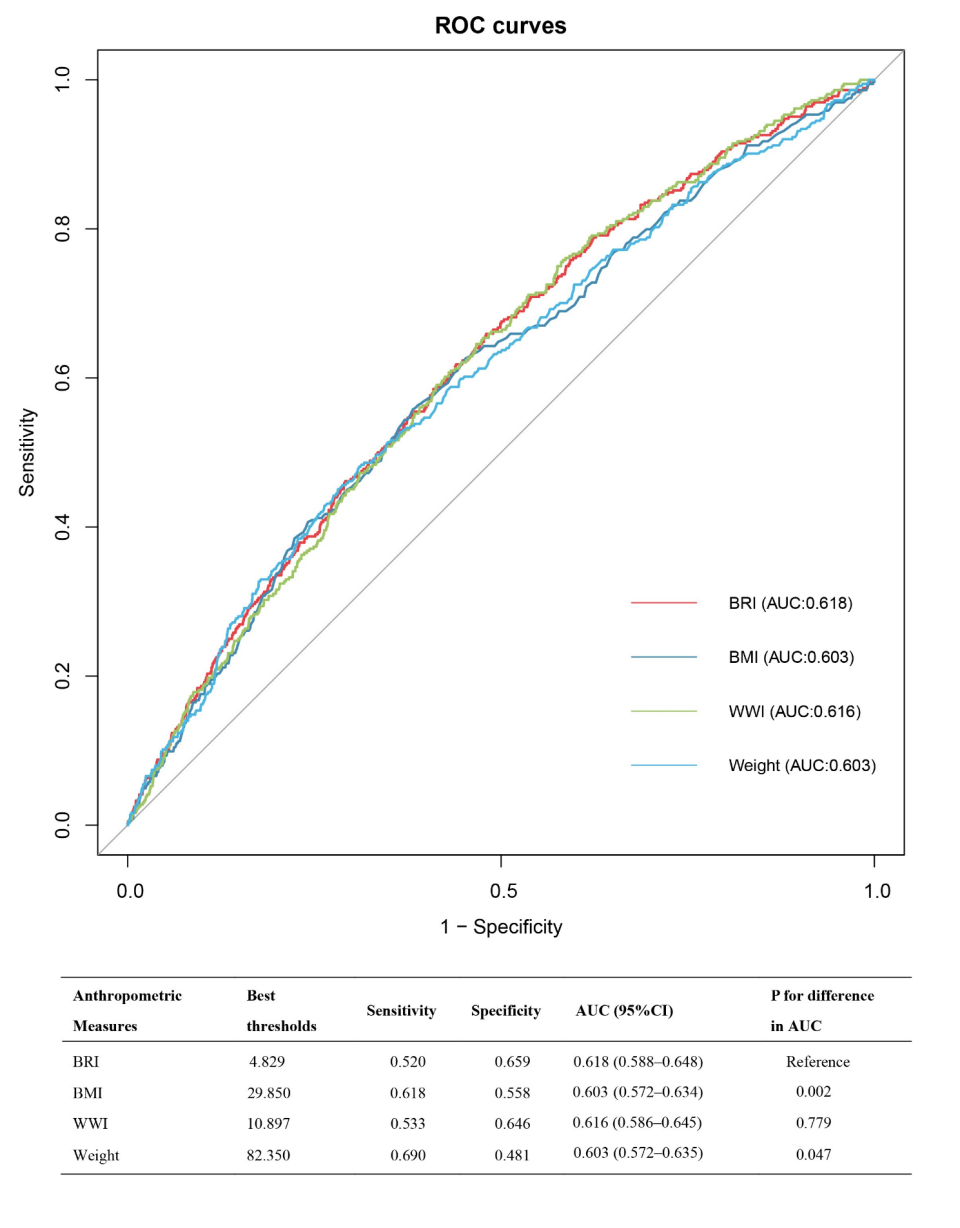
Receiver operating characteristic (ROC) curve analysis for infertility

We compared the predictive ability of BRI and various body measurement indicators for infertility likelihood by calculating the area under the curve (AUC) (Fig. 4). In this analysis, BRI demonstrated a strong advantage over the other indicators (WWI, BMI, Weight) with an AUC of 0.618 (95% CI, 0.588–0.648).

### Sensitivity analysis

To ensure the robustness of the results, multiple imputation was performed for missing baseline data. The significant positive correlation between BRI and infertility was maintained (Table S2).

## Discussion

This study demonstrates a positive correlation between BRI and infertility prevalence. After adjusting for various confounding factors, the positive association remained significant. Subgroup analyses showed that the relationship between BRI and infertility was consistent across different subgroups. Additionally, the results from smooth curve fitting and multiple imputation sensitivity analyses were similar, further supporting our conclusions. These findings suggest that BRI may be a useful predictor of infertility risk.

To our knowledge, this is the first study to investigate the association between BRI and infertility. The prevalence of obesity is increasing globally, and adipose tissue releases various bioactive molecules that affect reproductive health through multiple pathways [21]. The limitations of BMI as a measure of obesity are well-known; it does not account for differences in visceral fat distribution among individuals. While CT and MRI are standard methods for assessing visceral fat, they are expensive and time-consuming [22]. A meta-analysis indicated that BRI outperforms BMI, waist-hip ratio (WHR), body shape index (ABSI), and body adiposity index (BAI) in predicting metabolic syndrome [23]. Metabolic syndrome is a complex condition, with abdominal obesity and/or insulin resistance (IR) being increasingly recognized as its core components [24]. Studies assessing the metabolic and endocrine characteristics of obese women have observed that decreased secretion of FSH and LH coexists with hyperlipidemia and hyperinsulinemia, leading to the concept of “neurometabolic syndrome.” This underscores the profound impact of obesity on female reproductive function [25].

The mechanisms underlying the relationship between BRI and infertility are multifaceted and complex. Firstly, obesity, particularly visceral obesity, can affect female reproduction through direct mechanisms that damage the luteal phase and indirectly influence the hypothalamic-pituitary-ovarian (HPO) axis, causing neuroendocrine changes [26]. Obesity may impair the HPO axis, with hyperlipidemia and hyperinsulinemia in obese women leading to insensitivity to hypothalamic GnRH secretion [25]. Additionally, a study simulating hyperinsulinemia and hyperlipidemia in non-obese women showed that elevated insulin and lipid levels can acutely suppress LH and FSH, providing a possible mechanism for the relatively hypogonadotropic hypogonadism observed in obesity [27]. Another cross-sectional study indicated that obesity can reduce LH pulse amplitude and significantly decrease FSH secretion [28]. Secondly, systemic oxidative stress is positively correlated with visceral fat accumulation [29]. Ovarian adipose tissue induces oxidative stress either through the catalytic activity of NADPH oxidase or dysfunctional mitochondrial oxidative phosphorylation, which can damage oocytes through various pathways [30]. Thirdly, obesity is associated with low-grade inflammation, predominantly occurring in visceral fat deposits. Elevated lipoprotein lipase (LPL) and higher free fatty acid (FFA) uptake in visceral fat may lead to inflammation due to nutritional overload in the microcirculation of visceral adipose tissue [31]. Chronic inflammation can impact reproduction by damaging folliculogenesis, altering blood coagulability, and impairing endometrial receptivity through oxidative stress [32].

Numerous studies have shown the impact of obesity on female reproduction, and weight loss interventions have been demonstrated to benefit reproductive outcomes. A retrospective cohort study of 14,213 patients indicated that cumulative live birth rates (CLBRs) decrease with increasing BMI, while weight loss is beneficial for improving overall CLBRs [33]. Conversely, a randomized controlled trial involving 379 patients found that preconception-intensive lifestyle interventions did not improve fertility or reproductive outcomes [34], possibly due to differences in sample size. Previous research on obesity and reproductive health has predominantly used the World Health Organization (WHO) Body Mass Index (BMI) classification, which accounts for 50–70% of the variance in fat mass among non-pregnant women [13]. Recently, the focus has shifted towards more detailed management of obesity, particularly abdominal or central obesity. Various indices have been established to estimate central or abdominal obesity, including neck circumference (NC), waist-hip ratio (WHR), lipid accumulation product (LAP), visceral adiposity index (VAI), and Chinese visceral adiposity index (CVAI) [35, 36]. Studies have linked visceral obesity with cancer [37], diabetes [38], and cardiovascular diseases [39]. As the first study to explore the relationship between BRI and infertility, our findings indicate a linear relationship between BRI and infertility prevalence. BRI, which better accounts for visceral fat distribution compared to BMI, may offer new insights into the management and treatment of women with infertility.

To date, this study is the first to investigate the relationship between BRI and infertility using the NHANES database. The large sample size is a significant advantage, and the findings remain robust after adjusting for numerous confounding factors, conducting subgroup analyses, and performing multiple imputation analyses. However, the study has several limitations. Firstly, as a cross-sectional study, it cannot establish a causal relationship between BRI and infertility, which necessitates further prospective studies. Secondly, although many confounding factors were adjusted for, the limitations of the NHANES database prevented the inclusion of some potential infertility confounders, such as anatomical causes of infertility, which may have a weaker association with BRI. In addition, PSM and adjusted logistic regression analysis have their own advantages. PSM improves the comparability between groups, but it may also affect the statistical power due to the reduction of sample size. We believe that the results of both methods provide important information for understanding the potential relationship between BRI and infertility. Furthermore, metabolism-related disorders such as polycystic ovary syndrome (PCOS) and endometriosis may have a significant impact on the development of infertility [40, 41]. Due to limitations of the NHANES database, we were unable to obtain detailed information on polycystic ovary syndrome and endometriosis, and therefore could not directly control for these potential confounders in our analyses. Underrepresentation or imbalance of polycystic ovary syndrome and endometriosis in the study group may therefore have some impact on the interpretation of the results, and we need to explore these factors further in future studies. Finally, this study failed to differentiate between specific causes of infertility, which may affect the generalizability of this study’s findings. Future studies should consider stratifying the different causes of infertility to better understand the impact of obesity on different infertility types.

## Conclusion

In conclusion, this study demonstrates a strong positive association between BRI and the prevalence of infertility, supporting the application of BRI in the risk assessment of infertility and the promotion of reproductive health.

## Electronic supplementary material

Below is the link to the electronic supplementary material.

Supplementary Material 1

## Data Availability

The study involved the analysis of publicly available datasets. The data can be accessed at the following URL: https://www.cdc.gov/nchs/nhanes/.
