# Supplementary material for: Higher body roundness index (BRI) increases infertility among U.S. women aged 18–45 years
Source: BMC Endocr Disord. 2024 Dec 18;24:266. doi: 10.1186/s12902-024-01799-8 (PMC11654071; doi:10.1186/s12902-024-01799-8)
Supplement: Supplementary file 1 — Supplementary Material 1 [file 12902_2024_1799_MOESM1_ESM.docx]

**Supplementary Material**

**Table S1.** Description of covariates

| Covariates | Description in NHANES |
| --- | --- |
| Age | Divided into three groups: 18-25 years old, 26-34  years old, >34 years old |
| Race | Mexican American, Non-Hispanic Black, Non-Hispanic White, Other Race |
| Educational level | Below high school, High School or above |
| Marital status | Yes: Married/Living with partner |
| PIR | Poor: <1.3; Not Poor:>=1.3 |
| Smoking | Smoking status was grouped into never smoker (defined as <100 cigarettes in a lifetime), current smoker (defined as ≥100 cigarettes in a lifetime), and former smoker (defined as ≥100 cigarettes and had quit smoking) |
| Drinking | heavy drinking (≥4 drinks/day for men, ≥3 drinks/day for women, or ≥5 days of drinking in a month),  moderate drinking (≥3 drinks/day for men, ≥2 drinks/day for women, or ≥2 days of drinking in a month),  mild drinking (≤2 drinks/day for men, ≤1 drink/day for women, and ≥12 drinks in a year),  and never-drinking (total number of drinks in a year <12, and dietary alcohol content of 0%) |
| Diabetes | Diabetes was defined as a history of previous diabetes, HbA1c level ≥6.5%, or fasting blood glucose level ≥126 mg/dL |
| Hypertension | The diagnostic criteria consist of self-reported hypertension history, the utilization of antihypertensive medication, a systolic blood pressure (SBP) ≥ 140mmHg, or a diastolic blood pressure (DBP) ≥ 90mmHg |
| High cholesterol | Participants were asked whether they had high cholesterol |

PIR, Ratio of family income to poverty.

**Table S2.** Sensitivity analysis using the multiple imputation data results.

| **BRI** | **Model 1 [OR (95% CI)]** | ***p-value*** | **Model 2 [OR (95% CI)]** | ***p-value*** | **Model 3 [OR (95% CI)]** | ***p-value*** |
| --- | --- | --- | --- | --- | --- | --- |
| Continuous (Per 1 unit increase) | 1.15 (1.09, 1.20) | <0.001 | 1.13 (1.07, 1.20) | <0.001 | 1.13 (1.06, 1.19) | <0.001 |
| Quartile |  |  |  |  |  |  |
| Q1 | 1 (ref.) |  | 1 (ref.) |  | 1 (ref.) |  |
| Q2 | 1.48 (1.00, 2.19) | 0.053 | 1.21 (0.81, 1.82) | 0.300 | 1.28 (0.85, 1.91) | 0.200 |
| Q3 | 2.08 (1.32, 3.26) | 0.002 | 1.69 (1.05, 2.72) | 0.033 | 1.73 (1.03, 2.89) | 0.038 |
| Q4 | 3.14 (1.95, 5.06) | <0.001 | 2.57 (1.53, 4.32) | <0.001 | 2.54 (1.50, 4.28) | 0.001 |
| *P for trend* | <0.001 |  | <0.001 |  | 0.001 |  |

Model 1: no covariates were adjusted.

Model 2: age, gender, education level, marital, PIR, and race were adjusted.

Model 3: age, gender, education level, marital, PIR, race, smoking, drinking, hypertension, diabetes, and high cholesterol were adjusted.

Abbreviation: BRI, body roundness index; PIR, Ratio of family income to poverty; ORs, odds ratios; CI, confidence interval.

**Table S3.** Baseline characteristics of all participants were stratified by infertility after PSM, weighted.

| **Characteristic** | **Overall**, N = 10,466,689(100%) | **No**, N = 4,740,611 (45%) | **Yes**, N = 5,726,079 (55%) | **P Value** |
| --- | --- | --- | --- | --- |
| **No. of participants in the sample** | 722 | 361 | 361 | **-** |
| **Age (%)** |  |  |  | 0.568 |
| *18-25* | 1,196,126 (11%) | 476,172 (10%) | 719,954 (13%) |  |
| *26-34* | 3,267,724 (31%) | 1,525,299 (32%) | 1,742,425 (30%) |  |
| *>34* | 6,002,839 (57%) | 2,739,139 (58%) | 3,263,700 (57%) |  |
| **Race (%)** |  |  |  | **0.005** |
| *Non-Hispanic White* | 5,879,498 (56%) | 2,294,163 (48%) | 3,585,335 (63%) |  |
| *Other* | 1,781,903 (17%) | 956,283 (20%) | 825,620 (14%) |  |
| *Non-Hispanic Black* | 1,502,855 (14%) | 796,922 (17%) | 705,934 (12%) |  |
| *Mexican American* | 1,302,434 (12%) | 693,243 (15%) | 609,190 (11%) |  |
| **Married/live with partner (%)** |  |  |  | **0.002** |
| *No* | 2,940,122 (29%) | 1,656,747 (36%) | 1,283,375 (23%) |  |
| *Yes* | 7,370,394 (71%) | 2,960,920 (64%) | 4,409,475 (77%) |  |
| **Education level (%)** |  |  |  | **0.038** |
| *Below high school* | 1,322,835 (13%) | 738,179 (16%) | 584,656 (10%) |  |
| *High School or above* | 8,987,681 (87%) | 3,879,488 (84%) | 5,108,194 (90%) |  |
| **PIR (%)** |  |  |  | 0.302 |
| *Not Poor* | 7,200,680 (73%) | 3,093,417 (71%) | 4,107,263 (75%) |  |
| *poor* | 2,672,400 (27%) | 1,271,380 (29%) | 1,401,020 (25%) |  |
| **Smoking (%)** |  |  |  | 0.303 |
| *Never* | 6,492,591 (62%) | 2,977,126 (63%) | 3,515,465 (61%) |  |
| *Former* | 1,765,019 (17%) | 896,103 (19%) | 868,916 (15%) |  |
| *Current* | 2,209,080 (21%) | 867,383 (18%) | 1,341,698 (23%) |  |
| **Drinking (%)** |  |  |  | 0.938 |
| *former* | 848,893 (8.4%) | 342,345 (7.5%) | 506,548 (9.1%) |  |
| *heavy* | 2,888,043 (29%) | 1,344,294 (30%) | 1,543,749 (28%) |  |
| *mild* | 2,589,116 (26%) | 1,148,937 (25%) | 1,440,180 (26%) |  |
| *moderate* | 2,610,360 (26%) | 1,171,661 (26%) | 1,438,699 (26%) |  |
| *never* | 1,153,647 (11%) | 541,486 (12%) | 612,161 (11%) |  |
| **Hypertension (%)** |  |  |  | 0.482 |
| *No* | 7,964,383 (76%) | 3,541,429 (75%) | 4,422,954 (77%) |  |
| *Yes* | 2,502,307 (24%) | 1,199,182 (25%) | 1,303,126 (23%) |  |
| **Diabetes (%)** |  |  |  | 0.703 |
| *No* | 9,019,207 (90%) | 4,143,685 (91%) | 4,875,521 (90%) |  |
| *Yes* | 953,910 (9.6%) | 412,697 (9.1%) | 541,213 (10.0%) |  |
| **High cholesterol (%)** |  |  |  | 0.248 |
| *No* | 8,729,085 (83%) | 4,061,256 (86%) | 4,667,829 (82%) |  |
| *Yes* | 1,737,605 (17%) | 679,355 (14%) | 1,058,250 (18%) |  |
| **BRI (mean (SD))** | 6.31 (3.05) | 6.32 (3.26) | 6.30 (2.86) | 0.605 |

Mean (SD) for continuous variables: the P value was calculated by the weighted linear regression model.

Percentages (weighted N, %) for categorical variables: the P value was calculated by the weighted chi-square test.

Abbreviation: PSM, Propensity score matching; BRI, body roundness index; PIR, Ratio of family income to poverty.

**Table S4.** Adjusted odds ratios (ORs) of BRI and infertility after PSM, weighted.

| **BRI** | **Model 1 [OR (95% CI)]** | ***p-value*** | **Model 2 [OR (95% CI)]** | ***p-value*** | **Model 3 [OR (95% CI)]** | ***p-value*** |
| --- | --- | --- | --- | --- | --- | --- |
| Continuous (Per 1 unit increase) | 1.00(0.93,1.07) | 0.960 | 0.99(0.92,1.07) | 0.850 | 1.00(0.92,1.09) | >0.9 |
| Quartile |  |  |  |  |  |  |
| Q1 | 1 (ref.) |  | 1 (ref.) |  | 1 (ref.) |  |
| Q2 | 1.19(0.71,1.97) | 0.510 | 1.30(0.76,2.21) | 0.330 | 1.38(0.79,2.43) | 0.250 |
| Q3 | 1.09(0.61,1.96) | 0.760 | 1.12(0.61,2.05) | 0.720 | 1.16(0.58,2.31) | 0.660 |
| Q4 | 1.31(0.73,2.35) | 0.350 | 1.34(0.74,2.44) | 0.320 | 1.60(0.84,3.05) | 0.150 |
| *P for trend* | 0.450 |  | 0.490 |  | 0.280 |  |

Model 1: no covariates were adjusted.

Model 2: age, education level, marital, PIR, and race were adjusted.

Model 3: age, education level, marital, PIR, race, smoking, drinking, hypertension, diabetes, and high cholesterol were adjusted.

Abbreviation: BRI, body roundness index; PIR, Ratio of family income to poverty; ORs, odds ratios; CI, confidence interval.
